# Entrapment of the Proximal Loop of a Double-J Stent in the Renal Parenchyma Following Percutaneous Nephrolithotomy: An Exceptionally Rare Complication

**DOI:** 10.7759/cureus.95346

**Published:** 2025-10-24

**Authors:** Jihad El Anzaoui, Ali Akjay, Najwa Jmil, Mohamed Lezrek

**Affiliations:** 1 Urology, Mini-Invasive Surgery, Robotic Surgery, Artificial Intelligence, and Pedagogical Innovations Laboratory, Sidi Mohamed Ben Abdellah University Faculty of Medicine, Meknes, MAR; 2 Urology, Military Hospital Moulay Ismail, Meknes, MAR; 3 Urology, Sidi Mohamed Ben Abdellah University, Meknes, MAR; 4 Urology, Moulay Ismail Hospital, Meknes, MAR

**Keywords:** double-j stent, minimal invasive and endourology, percutaneous nephrolithotomy (pcnl), renal stone surgery, thulium laser

## Abstract

The double-J ureteral stent is a widely used device in urology. It is considered a revolutionary invention due to its significant clinical benefits. However, its increasing and sometimes prolonged use has been associated with specific complications. The use of a JJ stent is recognized as a standard drainage method following percutaneous nephrolithotomy (PCNL), and its proper placement is essential to prevent complications. We report the first documented case, to the best of our knowledge, in the literature of the proximal renal loop of a double-J stent embedded within the renal access tract during PCNL, which necessitated surgical intervention.

## Introduction

The JJ ureteral stent plays a pivotal role in modern urology [1]. It provides several therapeutic benefits, including preservation of renal function, prevention of urinary extravasation - a potential source of infection and fibrosis - and reduction of the risk of post-traumatic or post-surgical strictures. Despite its widespread use, complications such as migration [2], fragmentation [3], and encrustation [4] have been frequently reported.

Entrapment of the proximal loop within the renal parenchyma, however, is an exceptionally rare and scarcely reported complication. This unusual situation in endourology often necessitates the use of improvised treatment methods. Herein, we describe our experience with a unique case of proximal stent loop entrapment within the renal access orifice created for percutaneous nephrolithotomy (PCNL), which rendered standard stent removal techniques ineffective.

Entrapment of the proximal loop within the renal parenchyma, however, is an exceptionally rare and scarcely reported complication. This unusual situation in endourology often necessitates the use of improvised treatment methods. Herein, we describe our experience with a unique case of proximal stent loop entrapment within the renal access orifice created for percutaneous nephrolithotomy (PCNL), which rendered standard stent removal techniques ineffective. To the best of our knowledge, this is the first documented case in the literature describing the migration of a double J stent into the tract of a previous PCNL.

## Case presentation

A 14-year-old male presented with a chronic history of hematuria and left renal colic. A CT scan revealed two left renal stones: a 2 cm stone located in the renal pelvis and a 6 mm stone in the inferior calyx. These stones were managed using a supine split-leg modified percutaneous nephrolithotomy (PCNL) via a mid-calyceal approach. A 24 Ch tract was performed.

The procedure was uneventful, and complete stone clearance was achieved. No damage to the calyceal mucosa was noted. The procedure was concluded with retrograde insertion of a JJ stent under fluoroscopic guidance, with visual confirmation by nephroscopy through the Amplatz sheath.

One month postoperatively, an attempted stent removal failed due to firm adherence of the stent to the excretory system.

A flexible ureterorenoscopy using a 7.5 Ch scope, without an access sheath and under general anesthesia, was performed. Exploration revealed entrapment of the proximal coil within the renal parenchyma. The stent was entrapped at the site of the previous percutaneous tract in the middle calyx.

This unusual situation was managed by sectioning the mobile portion of the stent with a thulium laser. The proximal part of the stent was abandoned, and a new double-J stent was inserted. 

**Figure 1:**
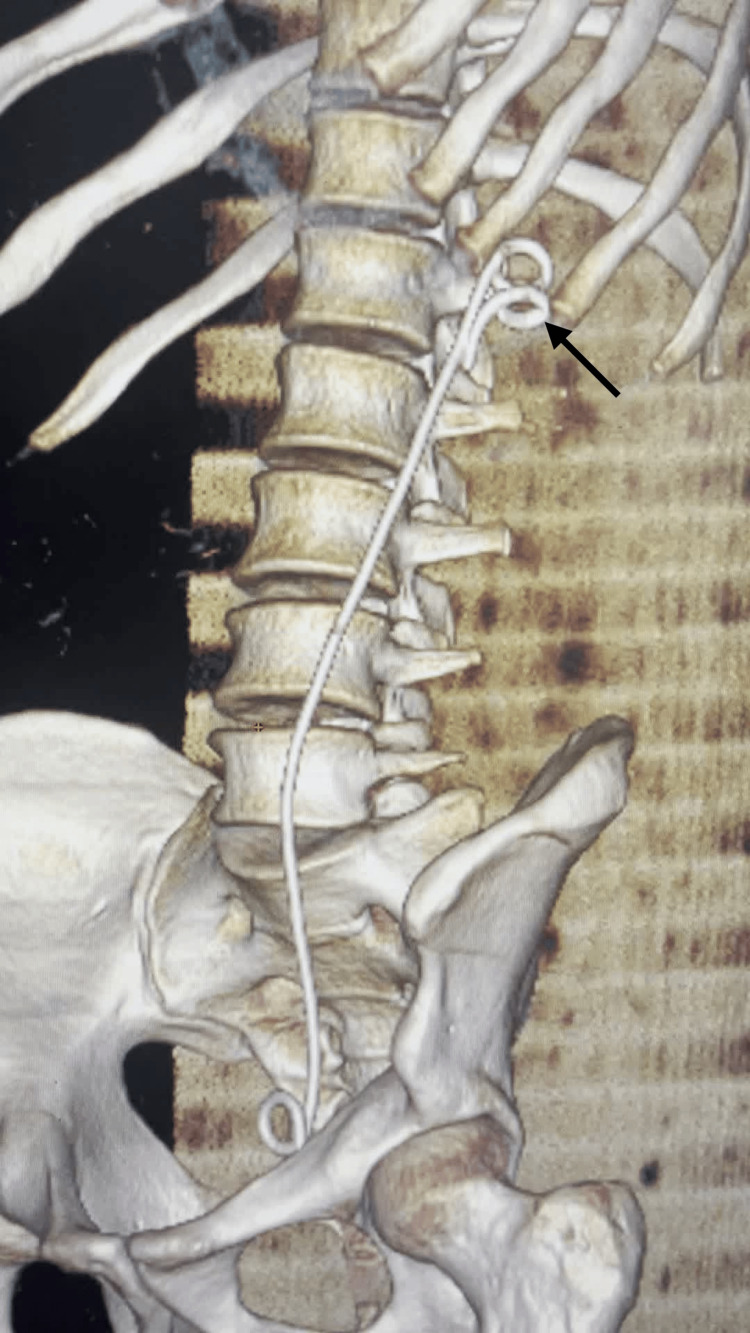
CT reconstruction showing a proximal loop of double-J stent (black arrow) abandoned into the renal parenchyma The proximal loop of the double-J stent was found to be embedded within the renal parenchyma during endoscopic exploration. The mobile portion of the stent was sectioned using a laser. Follow-up CT imaging showed the proximal loop remained in place, totally embedded within the renal parenchyma, while urinary drainage was maintained by the insertion of a new stent.

A postoperative abdominal CT confirmed that the proximal loop was totally embedded in the renal parenchyma (Figure 1). 

**Figure 2:**
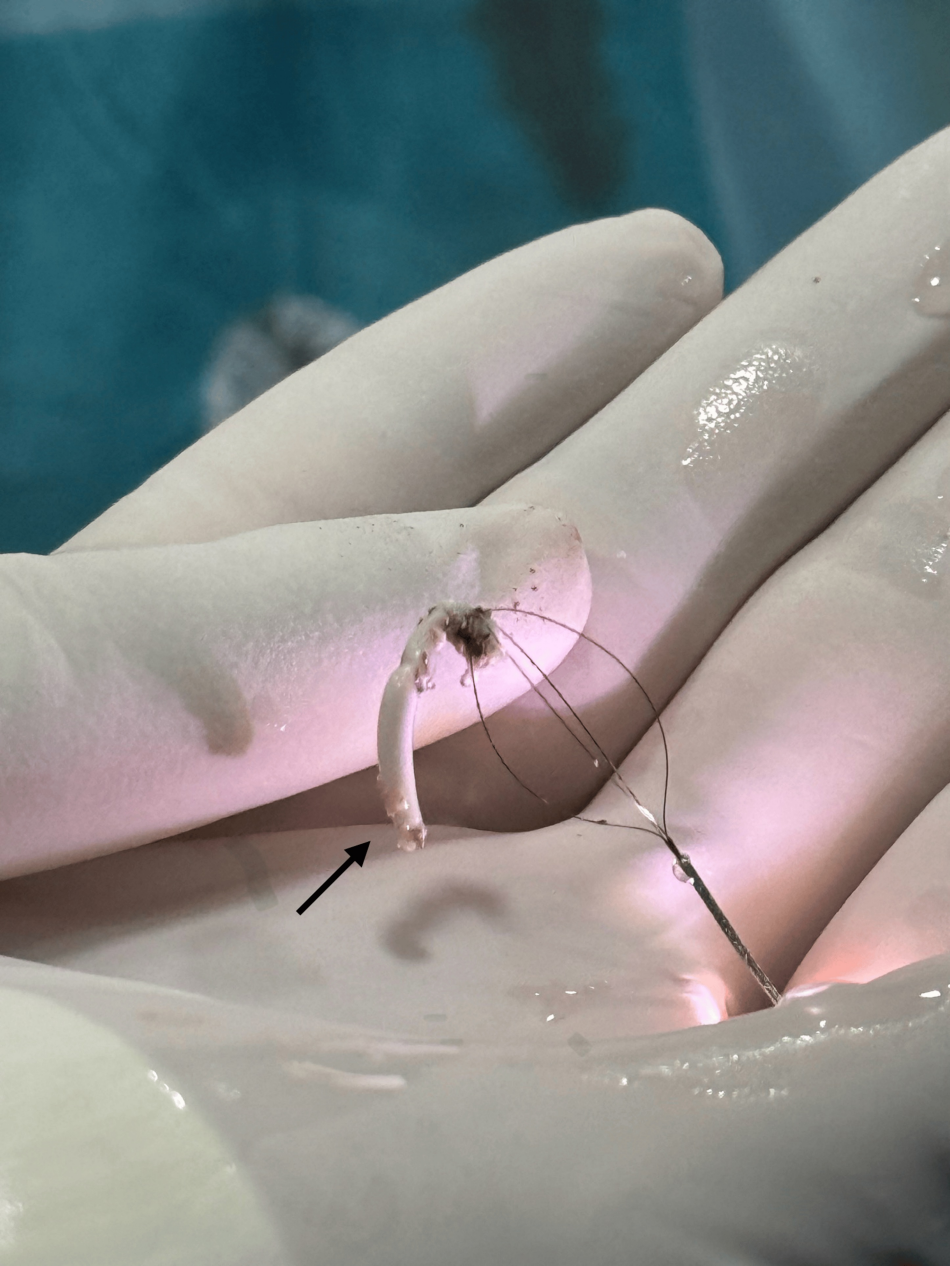
Fragment of the retained double-J stent (black arrow) extracted by endoscopic basket The retained segment was sectionned into several pieces and extracted using an endoscopic basket

**Figure 3:**
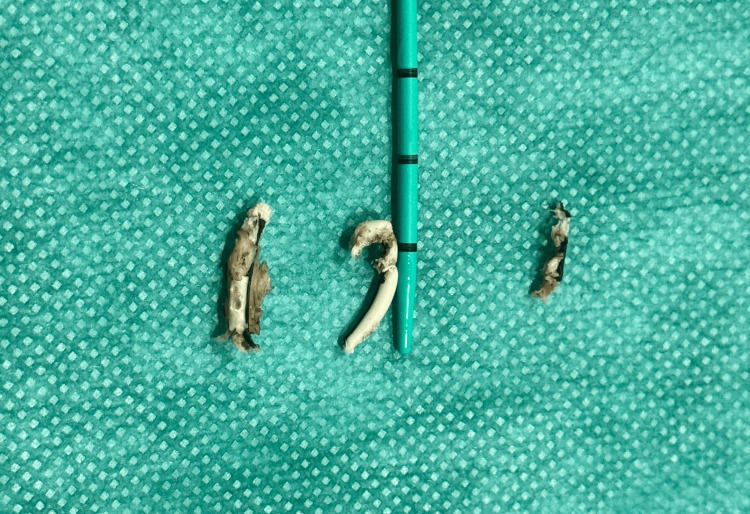
Extracted fragments of the retained part of the stent The retained segment was fragmented into three pieces, which were extracted using flexible ureteroscopy and endoscopic basket.

After one month, the patient underwent a second flexible ureterorenoscopy using a 7.5 ch device with a 10 Fr access sheath. A thulium laser incision of surrounding tissue (settings: 1 J, 15 Hz), followed by endoscopic basket retrieval (Figure 2), enabled successful removal of the embedded stent segment, which was extracted in several sectioned fragments (Video 1, Figure 3).

**Video 1:** Liberation of the retained segment from the surrounding tissue by using a thulium laser Adhesions to the surrounding tissue were incised using a thulium laser, with the incisions carried deep enough to free all retained segments.

## Discussion

This case illustrates a rare and poorly described complication. To our knowledge, only three similar cases have been reported in the literature [5,6]. In two cases [5], renal pelvis perforation during PCNL allowed migration of the stent loop into the perforation site, which was later encased by fibrotic tissue, preventing removal. Laser incision was required for extraction. In the third case, recently published by Abdelrasheed et al. [6], the authors report a case of proximal loop entrapment of a double-J stent. The authors hypothesized that the entrapment occurred at the site of the previous PCNL tract; however, they did not provide definitive evidence, such as endoscopic views or CT images, to confirm this. Therefore, we believe that their conclusion remains speculative.

In the absence of overt parenchymal perforation, antegrade stent placement itself may pose a risk. Specifically, if the JJ stent descends through the Amplatz sheath without nephroscopic visualization, a portion of it may remain extra-parenchymal, traversing the puncture site. Although this risk is theoretically known, it becomes significant when stent placement occurs blindly.

Visual and fluoroscopic guidance do not entirely prevent this complication, as secondary migration of the proximal loop into the parenchymal tract may still occur. This unusual positioning exposes the stent to adherence in the process of healing and fibrosis, rendering retrograde extraction impossible and necessitating surgical removal, which adds complexity to the management.

Unlike the cases reported by Hammad [5], our patient did not have an identified renal pelvis perforation. The placement of the double-J stent was confirmed under both fluoroscopic and direct visual control, suggesting that the entrapment was secondary to displacement into the tract of the initial PCNL puncture, which was the only site of parenchymal aggression. This displacement likely caused the stent to become entrapped within the previous puncture tract rather than a perforation site.

This emphasizes the importance of controlled stent placement and awareness that even with proper initial positioning, secondary displacement can occur, especially in the context of the PCNL.

We used laser incision to free the encased portion of the stent. This approach appears reasonable and safe, as the preoperative CT scan excluded any external extension of the loop or contact with adjacent vital structures. The parenchymal incision was feasible without significant bleeding, and the stent was subsequently extracted using an endoscopic basket.

To prevent entrapment of a portion of a double-J stent, five key factors should be considered. First, precise placement must be confirmed using fluoroscopic guidance or direct endoscopic visualization to ensure both coils are correctly positioned [7]. Second, timely stent removal is essential, especially when displacement occurs or prolonged indwelling time risks fibrosis and tissue ingrowth [8]. Third, selecting the appropriate stent length, design, and material can influence anchoring behavior and help prevent migration [9]. Fourth, in cases of distorted anatomy or pelvic injury, nephrostomy drainage may be a safer alternative to double-J stenting [10]. Lastly, minimizing the diameter of the transparenchymal tract during PCNL and reducing trauma to the excretory system can decrease the risk of stent migration and encasement into renal parenchymal injuries. Close monitoring and follow-up remain crucial to prevent complications associated with stents.

​This approach balances technical precision with patient-specific anatomical considerations, aiming to optimize outcomes and minimize risks related to double-J stent entrapment.

## Conclusions

Entrapment of the proximal loop of a double-J stent in the renal parenchyma following PCNL is an uncommon but significant complication. Preventive strategies include direct visual placement, limiting tract size, considering nephrostomy drainage, and planning for early stent removal when feasible. Awareness of this potential complication is essential for urologists performing PCNL. In cases of a completely embedded portion, laser extraction appears to be a reasonable method to free the stent segment.

## References

[1] Lee J, Katz M, Shah O (2021). Developments in ureteral stent technology. Front Surg.

[2] Mutou T, Kira S, Furuya R, Mochiduki T, Sawada N, Mitsui T (2023). A case of proximal migration of a double-J ureteral stent in a patient with metastatic gastric cancer. J Surg Case Rep.

[3] Al-Hajjaj M, Alam OA, Abu-Hussein B, Muhammad Al Husein HA (2022). Forgotten double-J ureteral stent: an analysis of 25 cases in a tertiary hospital. Ann Med Surg (Lond).

[4] Geavlete P, Georgescu D, Mulțescu R, Stanescu F, Cozma C, Geavlete B (2021). Ureteral stent complications - experience on 50,000 procedures. J Med Life.

[5] Hammad FT (2020). Embedded upper end of double J stent at the site of renal pelvis injury following percutaneous nephrolithotomy: a rare complication. BMC Urol.

[6] Abdelrasheed A, Sale A, Iqbal M (2025). Retention of a double-J stent upper coil in the renal parenchyma following percutaneous nephrolithotomy: a case report. Cureus.

[7] Ando T, Kazama A (2021). Double J stent migration as renal penetration. Urol Case Rep.

[8] Wang X, Ji Z, Yang P, Li J, Tian Y (2024). Forgotten ureteral stents: a systematic review of literature. BMC Urol.

[9] Şendoğan F, Turan T (2019). An exploration of factors that cause the pontaneous migration of double-j stents after retrograde intrarenal surgery. Erciyes Med J.

[10] Ghasemi-Rad M, Trinh K, Wynne D (2025). Nephrostomy (PCN) versus nephroureteral stent (double JJ): an ongoing battle. Urologia.

